# Analysis of Ambergris

**Published:** 1804-02-01

**Authors:** Bouillon Lagrange


### Analysis of Ambergris. *By* Bouillon Lagrange.

It is now almost generally admitted that ambergris is found in the stomach of the cachelot, called by naturalists *Physeter macrocephalus,* and that it seems to be the product of its digestion.

Dr. Swediaur has proved in his Researches on the Nature and Origin of Ambergris, that the beaks of the sepia, with which large pieces of ambergris, both those found on the coasts or at the surface of the sea, and those taken from the bellies of these whales, are mixed, belong to that species to which Linnæus has given the name of *Sepia octopoda.* The existence of these beaks and of other foreign bodies in ambergris is a convincing proof that it has been once in a state of softness or liquidity. M. Swediaur says, that the kind of whale which contains ambergris in its belly is that species from which spermaceti is extracted, which seems to be the *Physeter macrocephalus* of Linnæus, and which feeds chiefly on the large species of sepia. It is in the intestinal canal of this whale that the ambergris is found; to the animal it is a source of disease; this matter, when it issues from the bag which contains it, gradually acquires that solidity which it is observed to possess.

Ambergris is found in the Indian seas, near the Moluccas, the Maldives, and Madagascar, and on the coasts of China and Japan, and from Iola to the Manillas. It is often picked up on the Coasts of Maragnon, or of Brasil, but more commonly on those of Africa towards Cape Blanc, the Gulf of Arguin, the Bay of Portendia, and in some

 other other islands which extend from that of Mosambique to the Red Sea.

According to the accounts of several travellers, the inhabitants of the isles of Sanballat search for it in a very singular manner. After storms they proceed along the shore, and if there be ambergris on it they perceive it by the smell. There are certain birds and other animals on these shores which are fond of ambergris; and as they discover it at a distance by the smell, they go in search of it in order to eat it.

There can be no doubt that ambergris is an animal production. Several substances approach very near to it in smell, such as the excrements of some of the mammalia, and particularly those of oxen and pigs. I found that cows' dung dried in the sun has an odour very analogous to ambergris, and even to musk; and hence the name of *indigenous musk* given in some countries to this substance, prepared in this manner.

Ambergris *(ambrea grisea)* is a light substance which floats on the water, solid, opake, of an ash colour, veined with white and yellowish-brown, slightly odoriferous, but the odour of which develops itself as it grows old, or when it is mixed with musk or other aromatous substances, as it is prepared for perfumes or smelling waters.

Good ambergris in its natural state may be known if when scraped with the blade of a knife it adheres to the knife like wax, if it retains the impression of the nails and that of the teeth, and if, when pricked with a hot needle, it emits a fat odoriferous juice. Though solid, and in general brittle, it is not sufficiently hard to bear polishing, but rubbed with the nail it becomes smooth like hard soap.

Geoffroy, Neumann, Grim, and Brow, have classed ambergris among the bitumens. The analysis made of it by chemists is not sufficient to determine the nature of this substance. Ambergris fuses in the fire, says Geoffroy, into a resin of a yellow or gold colour; when applied to a flame it kindles and burns; spirit of wine does not dissolve it entirely, there remains a black matter like pitch on which it has no action. When dissolved it leaves some time after a white nebulous sediment, which gradually coagulates and becomes thicker and thicker; this *coagulum* being dried changes into a brilliant foliated earth, and which is not different from spermaceti.

In distillation, according to the same chemist, ambergris gives first an insipid phlegm, then an acid liquor or spirit, and a highly odorous yellow oil, with some portion of acid and and volatile dirty salt; in the last place there remains at the bottom of the retort a brilliant, black, and bituminous matter. It is here seen that this analysis, which does not differ from that given by all chemists, deserves to be re-examined, in order to give us correct ideas respecting the nature of this singular substance.

I think it my duty to acquaint those who may be desirous of repeating these experiments, to be very careful in their choice of the ambergris. Several varieties are common in commerce, the price of which establishes different species. There is no doubt that this substance is fabricated as *castorium* is made in some countries of Germany. Bayen assured me that he saw it made at Franckfort. This father of chemistry found that his memory did not deceive him; and, what is rare among travellers, he told the truth.

I have examined several kinds of ambergris used in commerce. Some of them vary in specific gravity, have a colour more or less dark, with very little odour, and are flexible; others are of an ash-grey colour, pretty hard; in the last place, others are almost stony, are scarcely soluble in alcohol, and have no odour.

The ambergris which I analysed was not purchased. I compared it with that in the cabinet of the Museum of Natural History, and found no difference either in the co-our or odour.

### *Physical Properties.*

It is of an ash-grey colour, interspersed in the inside with some yellow striæ; has a sweet mild odour, and grows soft between the fingers. When reduced to a fine powder it acquires a darker colour; pounded in a glass mortar it becomes agglutinated and adheres to the pestle.

Its taste is dull and almost insipid, and when put between the teeth it exhibits the same phenomena as wax.

Its specific gravity is from 844 to 849: that of water being 1000.

According to Brisson the specific gravity of ambergris is 9263. The weight of the cubic inch 4 gros 58 grains; that of the cubic foot 64 pounds 13 ounces 3 gros 47 grains.

The specific gravity of blackish ambergris is 7803. The weight of the cubic inch 4 gross 3 grains; that of the cubic foot 54 pounds 9 ounces 7 gross 35 grains.

### *Chemical Properties.*

Exp. 1. Ambergris, when placed on burning charcoal, burns, and becomes entirely volatilized; it then leaves an agreeable odour.

If

If this combustion is effected more slowly in a platina crucible it fuses, emitting the same odour; that of a fat body is also distinguished.

Nothing remains in the crucible but a black greasy spot.

Fifty degrees of heat (Reaumur's thermometer) are sufficient to fuse it. A brown brilliant liquid is thus obtained.

At eighty degrees it is volatilized under the form of white vapours.

Exp. 2. The odour developed during its volatilization, having made me suspect the presence of an acid analogous to that of balsams, an experiment was made to ascertain it.

A bit of ambergris was placed in a porcelain capsule covered by a bell, in which was suspended a paper tinged with turnsole. The apparatus being placed on a sand bath, the temperature was raised to the degree necessary to volatilize the ambergris: the paper speedily assumed a red colour. Nothing was now necessary but to discover the nature of this acid; and with this view Scheele's process for extracting acid of benjamin was employed.

The product was examined, and left no doubt in regrad to its analogy.

Exp. 3. The analysis with the retort added nothing to the knowledge already acquired in regard to the nature of ambergris.

A gentle temperature makes it fuse; in a more elevated one it is decomposed, and a whitish acid liquor and a light oil soluble in part in alcohol, which gives it a yellow colour, pass over into the receiver. There remains in the retort a light and very voluminous charcoal.

Exp. 4. Ambergris floats on water, and does not suffer itself to be penetrated by that liquid cold; it acquires neither odour nor savour. Boiling water produces no alteration on it. At that degree the ambergris dissolves and appears under the form of a brownish oily liquid; a small quantity of a black matter insoluble in alcohol is separated. The filtered liquor had neither odour nor colour, and only a slight bitter savour.

It is only in the ratio of the temperature, then, that ambergris dissolves, since in proportion as it is lowered it is found to have the same properties.

Exp. 5. Acids in general have very little action on ambergris. It has not yet been possible by the aid of these agents to discover the constituent parts of this compound.

Weakened sulphuric acid makes it experience no change. If concentrated, it lays bare little of the oxyd of carbon.

The

The same phænomena are exhibited by the muriatic and oxygenated muriatic acids.

Nitric acid raised to 18 degrees, and distilled from off that substance, in a pneumonic apparatus, gives for result nitrous gas, carbonic acid, and azotic gas.

The latter arises, no doubt, from the decomposition of some animal matters accidentally mixed with the ambergris, as may be observed in the examination of some fragments of it.

There is found in the retort, after the elastic fluids are extracted, a thick liquid inclining to yellow: when brought to a soft consistence the matter swells up a little, and when evaporated to dryness in a porcelain capsule there remained a dry brittle matter of a golden-yellow colour, brilliant and transparent, which exhibited characters analagous to resins.

Exp. 6. Alkalies unite with ambergris, and form with it soluble soaps.

Thirty grains of ambergris with ten grains of pure potash were put into a platina crucible and exposed to a gentle heat: the mixture fused without manifesting the presence of ammonia: by cooling, a brownish homogeneous mass was obtained.

One ounce of distilled water being poured over, it dissolved a part. This liquor was exceedingly alkaline.

The other portion not soluble remained under a soft tenacious form, which when warm adhered to the fingers.

A larger quantity of water being added, the whole was dissolved.

Caustic potash triturated some time in a mortar with ambergris does not facilitate its solution by water.

Ammonia cold has no action on it, but with the help of heat dissolves it. The mixture gradually becomes brown, and by evaporation gives a glutinous saponaceous matter, similar in every thing to that obtained by potash.

Exp. 7. Fixed oils, such as that of colsa, olives, &c. dissolve it with the help of heat in a very little time: the solution is yellow and transparent, and by evaporation becomes brown.

Exp. 8. Volatile oils also dissolve ambergris. Those of turpentine, savin, and hysop, do the same. With the help of heat the solution takes place speedily.

Evaporation gives a thick red magma which cannot be entirely dried, which burns on coals, emitting a thick smoke and an odour approaching that of ambergris. Alcohol dissolved this substance, and acquired a golden yellow colour: it was precipitated from it by the help of heat.

A complete solution cannot be obtained in volatile oils which are too old, even with the assistance of long continued heat.

Exp. 9. Solution by ether is very speedy, even without heat.

Exp. 10. The solution of ambergris by alcohol is that alone which can conduct to any certain results. The constituent parts may be separated in such a manner, that by uniting them a compound will be obtained, the characters of which approach near to those of the compound.

One gros of ambergris being reduced to fine powder and put into a phial, two ounces of rectified alcohol were poured over it. Twenty-four hours' maceration were sufficient to give to the alcohol a dark yellow colour. When the liquor had been filtered a new quantity of alcohol was added to the undissolved part, and the solution was facilitated by elevating the temperature. When the whole was dissolved, except a small quantity of black matter, the liquor was filtered still warm. It passed through clear; but by cooling there was separated a light pale yellow substance, a part of which remained attached to the sides of the vessel.

The first alcoholic tincture made cold, and that arising from the matter precipitated, were mixed, and evaporated to the consistence of extract: it was of a reddish yellow colour, adhered to the fingers, had an agreeable odour and a sweet taste. The evaporation was continued to dryness: in this state the matter with a brilliant and transparent aspect became soft between the fingers, and burnt in the same manner as resins.

The experiment was repeated, to establish in a more positive manner the characters of these two substances.

For this purpose the ambergris was left, as before, to macerate in alcohol for twenty-four hours: it was then filtered, and a new quantity of alcohol was added to the residuum: the maceration was the same. This second liquor was less coloured than the former. A third dose of alcohol was poured over the undissolved part, but it was scarcely coloured. This little action of the alcohol on the residuum gave reason to believe that it was no longer soluble in that menstruum: but I soon was convinced of the contrary. I heated the mixture, and the whole matter was dissolved in a moment. Nothing remained but about four grains of a black powder, which was oxyd of carbon. The liquor was filtered warm, and by cooling there was precipitated pitated a whitish yellow glutinous matter, which was separated from the liquor.

This experiment proves the possibility of separating by the help of alcohol three very distinct products; the first soluble cold, the second soluble warm, and the third insoluble, which is separated in the form of dust.

To establish the characters of the two first substances, the alcoholic tincture made cold was evaporated to dryness. There remained in the capsule 22 grains of a brown dry matter, brilliant in the fracture, unalterable in the air, and which became soft in a gentle heat: fifteen degrees were sufficient to give it a tenacious and glutinous consistence; when placed on coals it was entirely volatilized. If this experiment be made in a silver spoon, the volatilization is effected with the same rapidity; it emits an aromatic odour, and leaves no carbonaceous residuum.

As I suspected that this substance might have some analogy with resin extracted from the *propolis* by C. Vauquelin, I made some comparative experiments with it. The following are the points in which they differ:

1st. It fuses much more slowly.

2d. It emits a thick odorous smoke, which in smell approaches near to that of honey.

3d. It swells up, and leaves a very voluminous charcoal.

In the last place, this first substance extracted from ambergris, which may be considered as a real resin, is soluble in alcohol, and may be precipitated by water. This tincture reddens turnsole paper; which still proves that the alcohol dissolves at the same time the benzoic acid previously found, either by burning the amber under a bell, or in treating it with lime.

Nothing now remains but to examine the product obtained by alcohol warm, after the resin has been extracted by maceration.

I have already said that there is separated from the alcohol by cooling, a substance which deposits itself in part, and which adheres to the sides of the vessel.

When separated from the liquor and properly dried, it remains light and somewhat voluminous; it breaks and moulders under the pressure of the finger, but soon after it extends itself and becomes soft by the heat: it has a lamellated texture if left to cool slowly.

It retains between its moleculæ a little water and alcohol, which are separated by keeping this substance some time in a state of fusion. When re-fused it is much less whi e than before, and no longer possesses the granulated texture it exhibited.

exhibited. In a word, I have found in it all the properties of *adipo-cira*; a substance which C. Fourcroy found in the fat matter of dead bodies, and of which he described the characters in a memoir printed in the eighth volume of the *Annales de Chimie*.

*Recapitulation.*

It appears, then, that we may conclude from these experiments:

1st. That ambergris is a compound substance which burns and is entirely volatilized.

2d. That when distilled alone there is obtained from it a liquor slightly acid, and an oil partly soluble in alcohol and of an empyreumatic odour.

3d. That by sublimation, or the process of Scheele, benzoic acid is extracted from it.

4th. That water has no action on the substance.

5th. That by help of the nitric acid a matter analogous to resins mixed with *adipo-cira* is separated from it.

6th. That concentrated sulphuric, muriatic, and oxygenated muriatic acid char it without dissolving it.

7th. That with alkalies it forms a saponaceous compound.

8th. That fixed and volatile oils, ether, and alcohol, are the true solvents of ambergris.

9th. That alcohol affords the means of separating its constituent parts in the following proportions:

| | Grammes. |
|---|---|
| Adipo-cira - - - - - - - | 2.016 |
| Resin - - - - - - - - - | 1.167 |
| Benzoic acid - - - - - - | 0.425 |
| Carbonaceous matter - - | 0.212 |
| | 3.820 |

